# Caregiver burden and psychosocial outcomes in owners of dogs with chronic paresis and paralysis compared with owners of healthy dogs

**DOI:** 10.3389/fvets.2026.1837756

**Published:** 2026-06-08

**Authors:** Janine Pryjmak, Yury Zablotski, Susanne K. Lauer

**Affiliations:** LMU Small Animal Clinic, Centre for Clinical Veterinary Medicine, Ludwig-Maximilians-Universität, Munich, Germany

**Keywords:** caregiver burden, chronic neurological impairment, companion animal welfare, dog owners, paralysis, paresis, veterinary caregiving

## Abstract

**Objective:**

This study aimed to assess whether owners of dogs with chronic paresis or paralysis experience higher caregiver burden and altered psychosocial functioning compared to owners of healthy dogs.

**Methods:**

Between August 2024 and April 2025, owners of dogs with chronic paresis/paralysis (>6 months) and healthy dogs were recruited via veterinary clinics, physiotherapy centers, university hospitals, and social media to complete an online 122-item questionnaire. The survey included lifestyle questions and validated instruments: Zarit Burden Interview (ZBI), Perceived Stress Scale, Centre of Epidemiologic Studies Depression scale, Generalized Anxiety Disorder 7-Item Scale, Quality of Life Enjoyment and Satisfaction Questionnaire. Descriptive statistics and group comparisons were performed using appropriate parametric or non-parametric tests in R.

**Results:**

The study included 67 owners of paretic/paralyzed dogs and 90 owners of healthy dogs (88.5% women; 29.3% aged 19–29). Owners of paretic/paralyzed dogs reported significantly higher ZBI scores (*p* < 0.001) and lower quality of life (*p* = 0.03). Caregiver burden was higher when the owner had the dog before onset of paresis/paralysis (*p* < 0.001), experienced physical health complaints (*p* = 0.002), or reported negative impacts of paralysis on home cleanliness (*p* < 0.001). Owners who perceived their dog's quality of life as mediocre to poor showed higher ZBI scores (*p* < 0.001). In contrast, owners who received positive feedback from their social environment had significantly lower caregiver burden (*p* = 0.005).

**Conclusion:**

Owners of dogs with chronic paresis and paralysis face significantly higher caregiver burden. Factors such as timing of ownership, owner health, domestic hygiene, perceived canine quality of life, and social support influence caregiver stress levels.

## 1 Introduction

Chronic motor im pairment in dogs alters not only the animal's physical capabilities but also the everyday lives of those who care for them. Such impairment may arise as a long-term consequence of diverse spinal cord disorders, including intervertebral disc disease, fibrocartilaginous embolism, traumatic vertebral injury, degenerative myelopathies, and neoplastic or inflammatory spinal disease (1, 2). Among these conditions, intervertebral disc disease represents one of the most common causes of paresis and paralysis in dogs, with an estimated incidence of approximately 27.8 cases per 10,000 dog-years in insured populations (1). In emergency settings, it is also the most frequently identified cause of acute paralysis in dogs (3). Management commonly requires prolonged hands-on care, including the use of assistive devices such as slings, wheeled carts or drag bags, as well as ongoing bladder and bowel management in patients with urinary or fecal incontinence (4, 5). In addition, caregivers are often responsible for regular repositioning, hygiene measures, and preventive strategies to reduce the risk of pressure-related skin lesions and decubital ulcers (5). Although mobility aids and structured care routines may improve functional capacity and perceived canine quality of life, they may simultaneously increase caregiving workload, time demands, and emotional strain (4, 6).

In contrast to episodic disease management or time-limited postoperative care, paresis and paralysis are typically associated with sustained functional dependence and the absence of a clearly defined endpoint. Owners may need to provide daily physical assistance for months or years, often in combination with lifting or supporting body weight, managing incontinence, and adapting the home environment. These sustained caregiving demands can affect caregivers' physical health, daily routines, social participation, and psychological wellbeing.

The concept of caregiver burden provides a useful framework for understanding the impact of long-term caregiving demands. Caregiver burden describes the multidimensional physical, emotional, and psychological strain associated with providing sustained care to a dependent individual (7, 8). In human medicine, caregiver burden has been extensively studied and is frequently associated with increased perceived stress, depressive symptoms, anxiety, and reduced quality of life of the caregiver, particularly in situations involving high levels of physical dependence and prolonged daily care (9). Caregivers of individuals with leg paresis or paralysis, including those affected by spinal cord injury, stroke, neuromuscular disease, or amyotrophic lateral sclerosis, report particularly high levels of caregiver burden (10–12). In these populations, burden is closely linked to the care recipient's degree of physical dependence, the amount of time required for daily assistance, and the caregiver's own physical and mental health (12–18). Importantly, caregiver burden in these contexts is driven less by the specific neurological diagnosis than by the cumulative demands of hands-on care, hygiene management, and assistance with activities of daily (19).

Within veterinary medicine, a growing body of research indicates that caregiver burden can also be substantial in owners managing chronic disease in companion animals. Clinically meaningful caregiver burden has been documented in owners of dogs with epilepsy, behavioral disorders, dermatologic disease, osteoarthritis, cancer, and age-related cognitive dysfunction (20–25). Across these conditions, caregiver burden has been shown to relate more strongly to treatment complexity, sustained daily care demands, and perceived responsibility than to objective measures of disease severity.

Despite this expanding literature, caregiver burden in owners managing dogs with chronic paresis or paralysis has received comparatively little systematic attention (26). In contrast to primarily medical chronic diseases, chronic motor impairment is often associated with continuous physical dependence, sustained hands-on care and in a subset of cases, bladder or bowel management, and hygiene-related challenges (4, 26). These features suggest that chronic paresis and paralysis are associated with a distinct pattern of caregiver demands within veterinary neurology that warrants focused investigation. The objective of this study was to assess caregiver burden and associated psychosocial outcomes in owners of dogs with chronic paresis or paralysis in comparison with owners of healthy dogs using an electronic survey. We hypothesized that caregiver burden would be significantly higher among owners of neurologically impaired dogs than among owners of healthy dogs, and that higher caregiver burden would be associated with greater psychosocial impairment, including increased perceived stress, depressive and anxiety symptoms, and reduced quality of life.

## 2 Materials and methods

### 2.1 Ethics and consent

The study protocol was approved by the Ethics Committee of the Faculty of Veterinary Medicine of the Ludwig-Maximilians-Universität München (approval number: AZ 409-02-08-2024; date: 02.08.2024). At the commencement of the survey, participants were furnished with a concise synopsis of the study and requested to affirm their consent with the privacy policy. The participants were subsequently presented with the inclusion criteria and requested to confirm their eligibility prior to proceeding.

### 2.2 Study type

A cross-sectional online survey study was conducted, incorporating validated and previously published scales to assess caregiver burden and psychosocial function.

#### 2.2.1 Reporting guidelines

The Checklist for Reporting Results of Internet E-Surveys (CHERRIES) (23) was utilized in the preparation of this manuscript (27).

#### 2.2.2 Design

The target population comprised owners of healthy dogs and dogs afflicted with chronic paresis or paralysis.

#### 2.2.3 Development and pretesting

The survey was developed based on findings from previous caregiver burden research (28–30). Evasys, an internet-based software designed for the administration and automated evaluation of standardized questionnaires, was used for survey design and distribution. Prior to release, the survey underwent pretesting by two groups: dog owners without a professional background in veterinary medicine and veterinarians or postgraduate veterinary professionals. Pretesting focused on survey functionality and plausibility.

#### 2.2.4 Recruitment and access to the questionnaire

An open survey was created and made publicly accessible via a web link or QR code. Recruitment was conducted primarily through online channels, particularly social media platforms. In addition, the survey was disseminated through flyers displayed at the Small Animal Clinic of the Ludwig- Maximilians-Universität, announcements on the clinic's homepage and social media posts. Further distribution occurred through other veterinary clinics and physiotherapists. No incentives (e.g. prizes) were offered for participation. Data collection was conducted between August 2024 and April 2025. The survey comprised 122 items for owners of dogs with chronic paresis or paralysis and 88 items for owners of healthy dogs and included a general section as well as several validated questionnaires.

Information collected on caregivers included age, gender, relationship status, number of caregivers, living situation (house, apartment with or without an elevator), professional background (human medicine, veterinary practice, other profession) and the presence of physical health complaints. Dog related variables comprised age, size, weight, sex, age at onset of paresis or paralysis, underlying cause, timing of adoption relative to symptom onset, degree of paresis or paralysis, presence of incontinence, occurrence of pressure-related skin lesions, reactions of the social environment, use and acceptance of assistive devices such as carts or drag bags, and owner-assessed canine quality of life. Dogs were categorized by size based on owner reported body weight: small (0–15 kg), medium (>15–25 kg), and large (>25 kg). The survey also assessed whether negative effects on household cleanliness were associated with caregiver burden. The complete survey instrument is provided in the Appendix.

#### 2.2.5 Participants

The study included German speaking dog owners who fulfilled eligibility criteria. Participants were required to have lived with their dog for a minimum of 6 months. For dogs in the paresis/paralysis group, inclusion required the presence of neurologic symptoms for at least 6 months. Owners of healthy dogs were eligible only if their pets had no history of illness during the preceding 6 months. Exclusion criteria comprised the presence of additional acute or chronic medical conditions in the dogs as well as ongoing mental health treatment in the owners.

#### 2.2.6 Measures

The measures used in this study were selected based on findings from the human caregiving literature and prior veterinary research on caregiver burden. All instruments have been widely applied in clinical or research settings and demonstrate established psychometric properties, including reliability and validity.

#### 2.2.7 Zarit burden interview

Caregiver burden was assessed using an adapted version of the Zarit Burden Interview (ZBI) (7). The original instrument comprises 22 items in which caregivers rate the negative effects of caring for a relative on a five point scale ranging from never to nearly always. A total score above 20 indicates significant burden, with higher scores reflecting increased levels of burden. The ZBI has demonstrated strong psychometric properties, including internal consistency (α = 0.9–0.91) and construct validity, as indicated by a correlation of *r* = 0.73 with other measures of caregiver burden, quality of life and depression (31). This version of the ZBI used for pet caregivers was adapted based on the study by Spitznagel et al. (28) and comprises 18 items. The cut off value for this measure is 18, with scores above this threshold indicating excessive caregiver strain (see Table 1). Items assessed caregiving-related strain, including stress associated with balancing pet care with other personal or professional responsibilities.

**Table 1:** Classification of caregiver burden based on the adapted Zarit Burden Interview (ZBI) score by Spitznagel et al. (28).

ZBI (adapted) score	Description
< 18	Normal
19–23	High average burden
24–33	Mildly elevated burden
34–43	Moderately elevated burden
>44	Severely elevated burden

#### 2.2.8 Perceived stress scale

The Perceived Stress Scale (PSS) is a widely used instrument for the assessment of perceived stress (32). This 10 item scale evaluates the extent to which individuals perceive their lives as unpredictable, uncontrollable, or overwhelming, using a five point response scale ranging from never to very often. Higher total scores indicate greater perceived stress. The PSS has demonstrated acceptable internal consistency, with reported Cronbach's alpha ranging from α = 0.68 to α = 0.78 (33). In addition, previous research indicates that the PSS captures reliable variance beyond the general perceived stress factor (33). Perceived stress was assessed using items addressing perceived lack of control and unpredictability in daily life, such as difficulties controlling important aspects of one's life.

#### 2.2.9 Center for epidemiologic studies depression scale

The Center for Epidemiologic Studies Depression scale (CES-D) is a commonly used instrument for the assessment of depressive symptoms (34). This scale comprises 20 items in which participants rate symptoms and manifestations of depression on a four point scale ranging from rarely or none of the time to all of the time. In adults, CES-D scores ≥16 indicate an elevated risk of clinically relevant depressive symptoms. The scale demonstrates acceptable convergent validity with clinical diagnoses of depression (*r* = 0.45), good internal consistency (Cronbach's α = 0.82), and moderate test–retest reliability (*r* = 0.52) (35). Depressive symptoms were assessed using items addressing core affective and cognitive features of depression, such as depressed mood and difficulties with concentration.

#### 2.2.10 Generalized anxiety disorder 7-item scale

The Generalized Anxiety Disorder 7-Item Scale (GAD-7) is a seven item instrument used to assess symptoms of anxiety. Participants rate the frequency of symptoms on a four point scale ranging from not at all to nearly every day (36). A score of 10 or higher has demonstrated a sensitivity and specificity greater than 0.80 for the clinical diagnosis of generalized anxiety disorder (36). The internal consistency of the scale across subgroups is high, with a reported Cronbach's alpha of 0.89 (37). Anxiety-related symptoms were assessed using items addressing core features of generalized anxiety, such as feelings of nervousness or being on edge.

#### 2.2.11 Quality of life enjoyment and satisfaction questionnaire – short

The Quality of Life Enjoyment and Satisfaction Questionnaire - Short (QLES-QSF) is a 16 item instrument that assesses quality of life by evaluating enjoyment and satisfaction across several domains of daily life, including mood, health, work and relationships (38). Higher scores on the QLES-OSF indicate better quality of life. The instrument has demonstrated strong internal consistency (α = 0.9) and test retest reliability (α = 0.93) (39). Negative correlations between the QLES-QSF and indices of depression and global improvement (*r* = −0.30– −0.54) further support the validity of this measure (38). Quality of life was assessed using items addressing subjective wellbeing, such as satisfaction with mood during the preceding week.

### 2.3 Data analysis

#### 2.3.1 Power analysis

Previous studies investigating caregiver burden in companion animal owners have shown that the adapted ZBI correlates with measures of depression, stress, quality of life, treatment plan difficulty and non billable owner contacts, with correlation coefficients ranging from *r* = 0.31–0.59 (28, 40). Based on a significance level of α = 0.05 and statistical power of π = 0.8, a conservative sample size of 64 participants was calculated to detect a medium effect size (*r* = 0.5). In anticipation of incomplete responses, a larger sample size was targeted through online recruitment.

#### 2.3.2 Statistical analysis

All statistical analyses were performed using R [version 4.2.1 (2022-06-23)]. Continuous variables included psychometric scores (e.g., ZBI, PSS, CES-D, GAD-7, QLES-QSF) and dog-related measures such as age and body weight, whereas categorical variables included demographic characteristics (e.g., gender, profession), as well as dog- and caregiving-related factors (e.g., incontinence status, use of assistive devices, and living situation). The distribution of continuous variables was assessed using the Shapiro–Wilk test. Homogeneity of variances was assessed using Levene's test with median centering where applicable. As most variables, including Zarit Burden Interview (ZBI), Perceived Stress Scale (PSS), Center for Epidemiologic Studies Depression Scale (CES-D), Generalized Anxiety Disorder-7 (GAD-7), Quality of Life Enjoyment and Satisfaction Questionnaire (QLES-QSF) scores and dog- and caregiver-related measures, deviated from a normal distribution, non-parametric methods were applied.

Group comparisons between owners of healthy dogs and dogs with chronic paresis or paralysis were conducted using Mann–Whitney *U*–tests for continuous variables and chi-square tests of independence for categorical variables. When contingency tables contained small expected cell counts (e.g., sparse categories such as widowed relationship status or rare assistive device constellations with *n* < 5), Fisher's exact test was used to obtain accurate *p*-values. For categorical variables with more than two levels, significant omnibus tests were followed by *post-hoc* pairwise proportion tests with Benjamini–Hochberg false discovery rate correction.

Within the subgroup of owners of dogs with chronic paresis or paralysis, associations between caregiver burden or quality of life and dog- or caregiver-related variables (e.g., caregiver age group, relationship status, profession, cause of neurologic symptoms, living situation, incontinence, use and acceptance of mobility aids, locomotion status) were examined using the Kruskal–Wallis test for multi-level predictors and Mann–Whitney *U*-tests for binary predictors. Significant Kruskal–Wallis tests were further explored using Benjamini–Hochberg-adjusted *post-hoc* pairwise comparisons.

Correlations between continuous psychometric outcomes (e.g., ZBI with PSS, CES-D, GAD-7, QLES-QSF; PSS with CES-D and GAD-7) were analyzed using Spearman's rank correlation coefficients. All statistical tests were two tailed, and a *p*-value below 0.05 was considered statistically significant after adjustment where applicable.

## 3 Results

### 3.1 Participant demographics

Demographic characteristics and group comparisons are summarized in Table 2.

**Table 2:** Sociodemographic and dog-related characteristics of the study population and group comparisons (owners of dogs with chronic paresis or paralysis vs. healthy dogs)

Variable	Paresis/paralysis group	Healthy group	*p*-value
Sample size (*n*)	67	90	
Female (%)	92.5	85.6	0.2
Veterinary profession (%)	11.9	28.9	0.002
Number of caretakers (1/2/3/4) (%)	37.3/46.3/9.0/7.5	26.7/53.3/8.9/10.0	0.89/0.06/0.59/0.29
Dog age (years), Mdn [95% CI]	8.9 [7.9-9.9]	5.6 [4.9-6.3]	< 0.001
Dog weight (kg), Mdn [95% CI]	15.5 [13.4-17.6]	21.6 [19.3-24.0]	< 0.001
Dog sex (female/female neutered/male/male neutered) (%)	22.4/25.4/16.4/35.8	27.8/20.0/33.3/18.9	0.11/0.87/0.003/0.27
Dog size (small/medium/large) (%)	50.7/41.8/7.5	25.6/37.8/36.7	0.23/0.53/ < 0.001

The study included 157 participants, the majority of whom were female (89.1%). Participants were predominantly single, with ages most frequently falling within the range of under 19 to under 29 years. A significantly higher proportion of owners of healthy dogs were employed in the veterinary field compared with owners of dogs with chronic paresis or paralysis (*p* = 0.002). Gender distribution and number of caregivers did not differ significantly between groups. Among owners of dogs with chronic paresis or paralysis, residential conditions included houses (53.7%), ground floor apartments (16.4%), apartments with elevator access (6.0%), and apartments without elevator access (23.9%).

In addition, owners of chronically paralyzed dogs were surveyed regarding the presence of any physical health complaints. Dogs in the healthy group were significantly younger than dogs with chronic paresis or paralysis (Mdn [range]: 5.0 [4.0–6.0] years vs. 9.0 [8.0–10.0] years; *p* = 0.001). The proportion of male dogs was significantly higher in the healthy group (*p* = 0.003) and intact male dogs were more frequent among healthy dogs than among dogs with chronic paresis or paralysis (*p* = 0.007). The study population included dogs of various breeds and sizes, indicating a heterogeneous sample (see Table 3). Median body weight was significantly lower in dogs with chronic paresis or paralysis (Mdn: 14.0 [11.0–16.0] kg) compared with healthy dogs (Mdn: 20.0 [17.5–24.0]; *p* < 0.001). A significantly higher proportion of owners of large healthy dogs participated in the study compared to owners of dogs with chronic paresis or paralysis (*p* < 0.001). Reported causes of neurological impairment included trauma (34.3%), intervertebral disc disease (31.3%), degenerative myelopathy (13.4%), unknown origin (11.9%), and miscellaneous causes (7.5%). Degrees of functional impairment varied and included standing only with support (43.3%), walking only with support (44.8%), independent but unsteady walking (11.9%); independent walking with frequent falls (7.5%), and complete absence of voluntary hindlimb movement with inability to stand or walk independently (49.3%). These categories were not mutually exclusive. Adoption occurred prior to the onset of neurological symptoms in 61.2% of dogs and after symptom onset in 38.8%. Owners reported urinary incontinence in 10.4% of dogs, fecal incontinence in 11.9%, combined urinary and fecal incontinence in 59.7%, and full continence in 17.9% of dogs. Among owners of chronically paretic or paralyzed dogs, 19.4% rated their dog's quality of life as poor to mediocre, while 35.8% assessed the quality of life as good and 44.8% as very good.

**Table 3:** Breed distribution of dogs by size category and study group (paresis/paralysis vs. healthy).

Size category (kg)	Breed	Paresis/paralysis	Healthy
Small (0–15)	Pug	8	0
	French bulldog	6	1
	Dachshund	1	2
	Mixed breed	14	7
	Other breeds^*^	5	13
Medium (>15–25)	Australian shepherd	1	1
	Border collie	1	2
	French bulldog	4	0
	Husky	1	1
	Labrador	2	1
	Magyar vizsla	1	1
	Mixed breed	13	15
	Other breeds^*^	5	13
Large (>25)	Bernese Mountain dog	0	2
	Boxer	1	1
	German shepherd	0	5
	Golden retriever	0	2
	Labrador	0	5
	Mixed breed	3	6
	Other breeds^*^	1	12

^*^Other breeds include purebred dogs represented by a single individual.

### 3.2 Zarit burden interview

**Figure 1:**
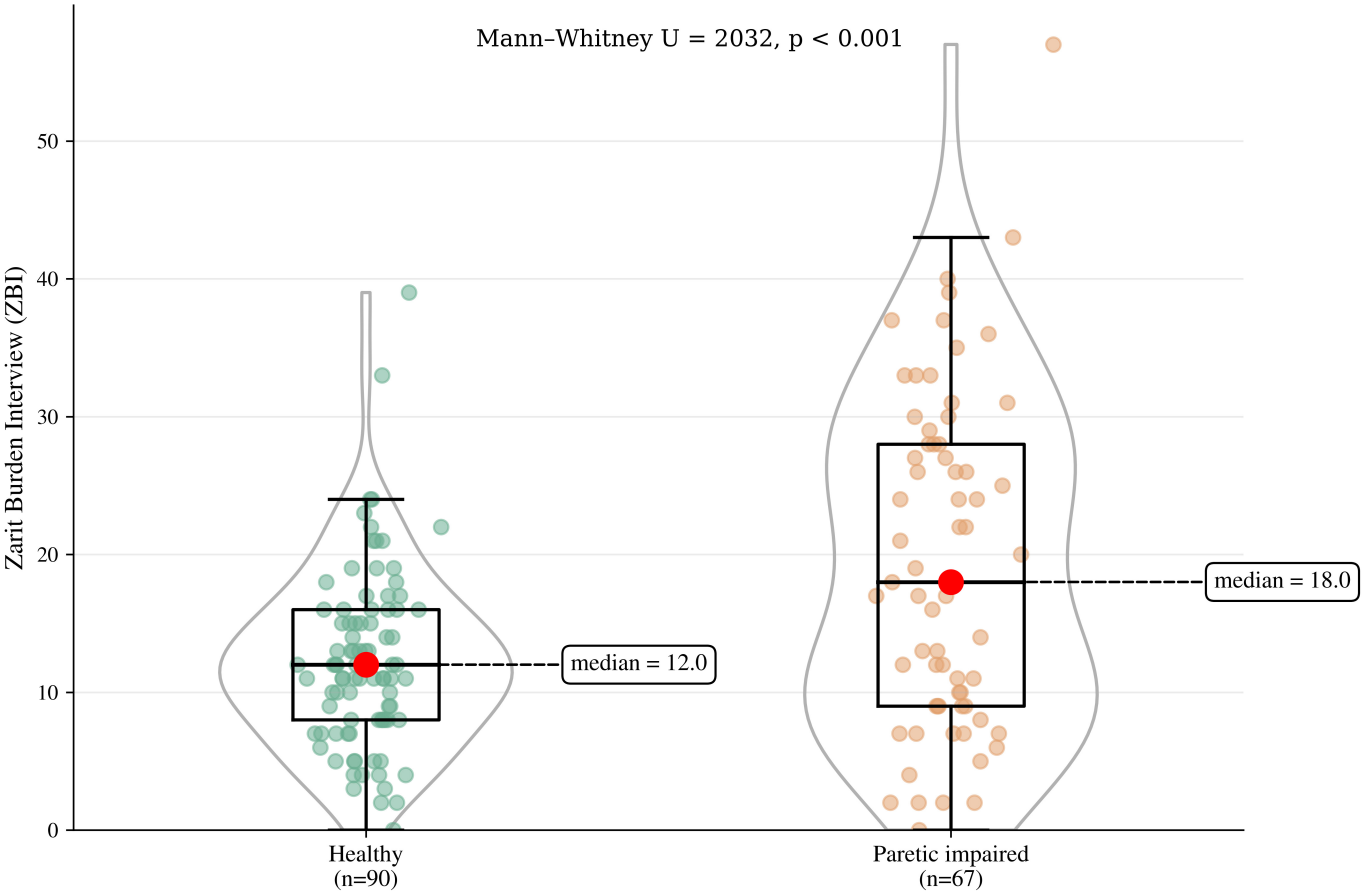
Caregiver burden in owners of healthy dogs and dogs with chronic paresis or paralysis. Violin and boxplots depict Zarit Burden Interview (ZBI) scores in owners of healthy dogs and owners of dogs with chronic paresis or paralysis. Points represent individual respondents, boxplots show the interquartile range with the median, and red dots indicate group medians.

Zarit Burden Interview (ZBI) scores were significantly higher in owners of dogs with chronic paresis or paralysis (Mdn [range]: 18.0 [12–24.0]) than in owners of healthy dogs (Mdn [range]: 12.0 [11.0–13.0]; (*p* < 0.001), see Figure 1). As higher ZBI scores indicate greater caregiver burden, with values above 18 reflecting elevated burden and increasing severity, the observed scores in the paresis/paralysis group correspond to a clinically relevant level of burden. Within the paresis/paralysis group, ZBI scores were not significantly affected by owner age, gender, relationship status or number of caregivers (all *p* > 0.05). Owners employed in veterinary medicine reported lower ZBI scores (Mdn [range]: 8.5 [2.0–16.0]) than owners employed in human medicine (Mdn [range]: 20.0 [9.0–28.0]) or in other professions (Mdn [range]: 21.5 [14.0–26.0]) (*p* = 0.03).

Among dog-related variables, significantly higher ZBI scores were observed in owners of older dogs (*p* = 0.02) and of dogs with a later age at onset of neurological impairment (*p* < 0.001). Owners who had adopted their dog prior to symptom onset reported significantly higher caregiver burden (Mdn [range]: 26.0 [20.0–29.5]; *p* < 0.001). In contrast, a longer duration of neurological symptoms was associated with lower ZBI scores (*p* = 0.04). Dog-related characteristics such as size (*p* = 0.35), body weight (*p* = 0.08), and sex (*p* = 0.77) were not significantly associated with caregiver burden.

**Figure 2:**
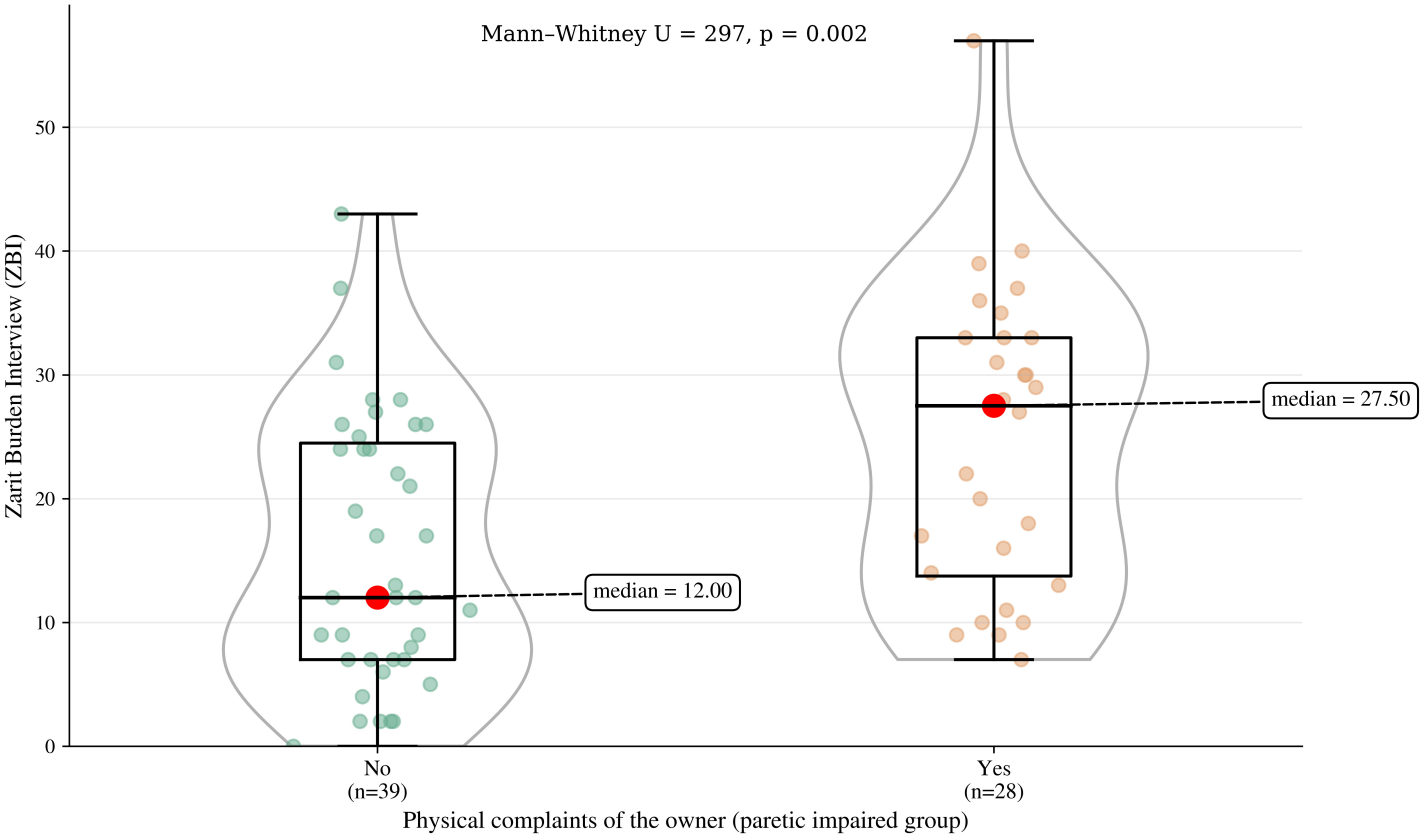
Caregiver burden by owner physical complaints in the paretic impaired group. Violin and boxplots display ZBI scores in owners of dogs with chronic paresis or paralysis stratified by the presence of owner-reported physical complaints related to caregiving (no vs. yes). Points represent individual respondents, boxplots show the interquartile range with the median, and red dots indicate group medians.

**Figure 3:**
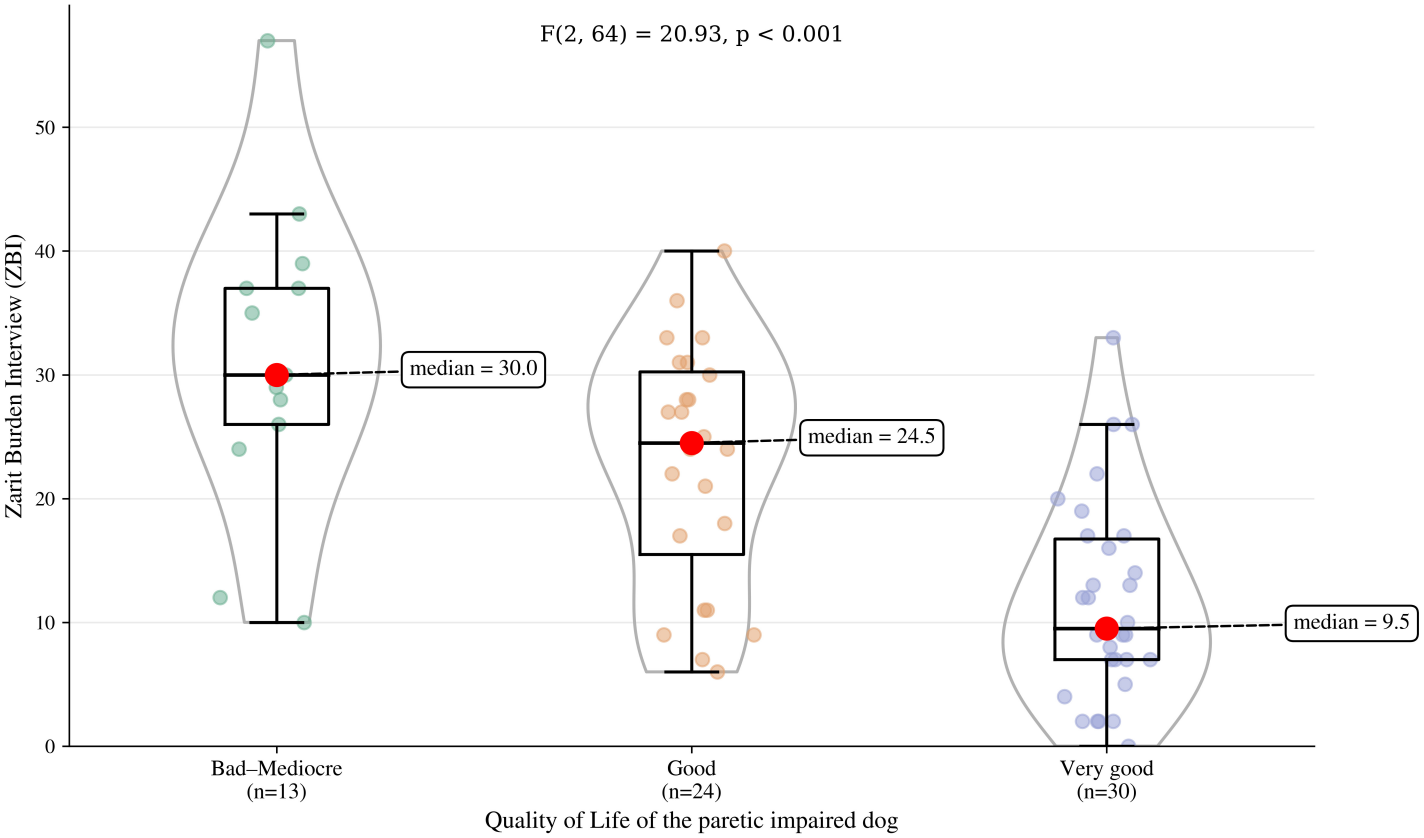
Caregiver burden by owner-rated quality of life of the paretic impaired dog. Violin and boxplots show ZBI scores in owners of dogs with chronic paresis or paralysis stratified by owner-rated canine quality of life (bad–mediocre, good, very good). Points represent individual respondents, boxplots indicate the interquartile range with the median, and red dots denote group medians.

Among caregiving-related factors, significantly higher burden was observed in owners reporting a strong negative impact of their dog's neurological impairment on household cleanliness (Mdn [range]: 29.5 [26.0–39.0]; *p* < 0.001), in owners reporting physical health complaints related to caregiving (Mdn [range]: 27.5 [17.0–31.0]; *p* = 0.002; see Figure 2) and in owners who rated their dog's quality of life as poor to mediocre (Mdn [range]: 30.0 [26.0–37.0]; *p* < 0.001; see Figure 3). Owners receiving strong positive reactions from their social environment reported significantly lower caregiver burden (Mdn [range]: 12.0 [9.0–17.0]; *p* = 0.009).

No significant associations were observed between caregiver burden and the degree of neurological impairment (all *p* > 0.05), type of incontinence (urinary, fecal, or combined; *p* = 0.64), use or acceptance of assistive devices such as carts or drag bags (all *p* > 0.05), or the presence of pressure-related skin lesions (*p* = 0.18). Details of all tested factors and their associations with ZBI scores are provided in Table 4.

**Table 4:** Associations between selected owner- and dog-related factors and caregiver burden (ZBI) in owners of dogs with chronic paresis or paralysis.

Factor	Median ZBI score [95% CI] - Yes	Median ZBI score [95% CI] - No	*p*-value
Ownership before paresis/paralysis	26.0 [20.0–29.5]	9.0 [7.0–12.0]	< 0.001
Owner has physical health complaints	27.5 [17.0–31.0]	12.0 [9.0–21.5]	0.002
Very bad-mediocre acceptance of carts	26.5 [12.0–35.0]	17.0 [11.0–22.0]	0.08
Drag bag use	9.0 [7.0–26.0]	19.0 [13.5–24.5]	0.30

### 3.3 Psychosocial outcomes

Owners of dogs with chronic paresis or paralysis reported significantly higher perceived stress scores than owners of healthy dogs (*p* = 0.04) and significantly lower quality of life scores on the QLES-QSF (*p* = 0.03). No significant group differences were observed for depressive symptoms as measured by the CES-D (*p* = 0.24) or for anxiety symptoms as measured by the GAD-7 (*p* = 0.38). Descriptive statistics for all psychosocial measures are presented in Table 5.

**Table 5:** Comparison of psychosocial outcomes between owners of dogs with chronic paresis or paralysis and healthy dogs.

Measure	Paresis/paralysis group	Healthy group	*p*-value
Perceived Stress Scale (PSS), Mdn [95% CI]	21.0 [20.0–22.0]	20.0 [19.0–21.0]	0.04
CES-D (depression), Mdn [95% CI]	6.0 [3.0–9.0]	5.0 [3.0–6.0]	0.24
GAD-7 (anxiety), Mdn [95% CI]	3.5 [2.0–5.0]	3.0 [2.0–3.0]	0.38
QLES-QSF (quality of life), Mdn [95% CI]	43.5 [39.5–46.0]	46.0 [44.0–47.0]	0.03

Caregiver burden was positively correlated with perceived stress (PSS; *p* < 0.001), depressive symptoms (CES-D; *p* < 0.001), and anxiety symptoms (GAD-7; *p* < 0.001), and negatively correlated with quality of life (QLES-QSF; *p* < 0.001) (see Table 6).

**Table 6:** Spearman's rank correlations between caregiver burden (Zarit Burden Interview, ZBI) and psychosocial outcomes.

Psychosocial measures	Spearman's	*p*-value
Perceived Stress Scale (PSS)	0.46	< 0.001
Center for Epidemiologic Studies Depression Scale (CES-D)	0.74	< 0.001
Generalized Anxiety Disorder 7-Item Scale (GAD-7)	0.64	< 0.001
Quality of Life Enjoyment and Satisfaction Questionnaire – Short (QLES-Q-SF)	−0.73	< 0.001

## 4 Discussion

Chronic motor impairment in dogs alters not only mobility and independence, but also the daily structure, physical workload, and emotional landscape of those who provide long-term care. The objective of this study was to assess caregiver burden and associated psychosocial outcomes in owners of dogs with chronic paresis or paralysis compared with owners of healthy dogs. In accordance with the study hypotheses, caregiver burden was significantly higher among owners of neurologically impaired dogs. Furthermore, higher caregiver burden was strongly associated with greater psychosocial impairment, including increased perceived stress, more pronounced depressive and anxiety symptoms, and reduced quality of life. Although group differences in depressive and anxiety symptoms were not statistically significant, caregiver burden demonstrated robust correlations with all psychosocial measures. Overall, the primary hypotheses of this study were supported.

This study indicates that caregiver burden in owners of dogs with chronic paresis or paralysis is more strongly related to everyday caregiving demands than to neurological severity. In the present sample, higher burden was predominantly associated with practical challenges, including hygiene-related tasks, physical strain during caregiving, and a lower owner-rated quality of life of the dog. In contrast, clinical characteristics such as the degree of paresis, the presence of urinary or fecal incontinence, the use of assistive devices and the presence of pressure-related skin lesions were not significantly associated with caregiver burden. Comparable patterns have been reported in human caregiving research, in which caregiver strain is influenced less by objective disease severity and more by the cumulative demands of daily care, particularly tasks involving hygiene and physical assistance (41–43). The strong association between reported negative effects on household cleanliness and higher caregiver burden suggests that perceived disruption of the domestic environment may represent a particularly salient aspect of the caregiving experience. While the present study did not quantify specific care tasks or time investment, this finding indicates that subjective impact on everyday living conditions may be more closely related to burden than clinical severity parameters.

Within veterinary medicine, elevated caregiver burden has been described in owners of dogs and cats with chronic or terminal conditions, including cancer, internal medicine disorders, and other progressive diseases requiring ongoing treatment and routine adjustments (28, 40). In particular, treatment-related routine changes and the perception that disease management imposes ongoing practical constraints have been linked to elevated burden (40). Qualitative work in canine epilepsy further highlights how sustained care responsibilities alter daily routines, restrict social participation, and increase emotional strain, even when objective medical parameters vary (44). In contrast, caregiver burden did not differ significantly between owners of dogs with limb amputation and owners of healthy dogs, indicating that the presence of a medical condition alone is not sufficient to increase caregiver burden. Instead, factors such as limitations in shared activities, use of assistive devices, and social feedback were more closely associated with caregiver experience (45). Together, these findings suggest that caregiver burden is shaped less by diagnosis itself than by the extent to which a condition affects daily life. This pattern is also reflected in the present study, where caregiver burden in chronic neurological impairment was more closely related to perceived daily-life impact than to neurological grading. Consistent with this interpretation, supportive reactions from the social environment were associated with lower caregiver burden in the present study. This finding is consistent with evidence from both human and veterinary caregiving research identifying perceived social support as a protective factor in long-term caregiving situations (46–52).

The human-canine bond may influence caregiver burden in chronic disease contexts, although its effects appear to be context-dependent rather than uniformly protective (53, 54). In owners of dogs with cognitive dysfunction, stronger emotional closeness has been associated with increased anticipatory grief, which in turn correlates with higher caregiver burden (53). Illness-related changes may further intensify bond-related processes, including perceived responsibility, worry, and grief, while altering relationship dynamics and caregiving behavior (55, 56). Stronger owner–dog relationships have also been associated with increased veterinary care-seeking and challenges in decision-making, particularly in end-of-life contexts (55). In addition, owner-perceived canine welfare and behavioral changes have been identified as potential mediating factors linking attachment to caregiver outcomes (57). As the human-canine bond was not assessed in the present study, its role as a potential modifier of caregiver burden remains to be determined.

Caregiver burden was higher when neurological impairment developed later in the dog's life or when owners had adopted the dog prior to symptom onset, whereas longer duration of neurological symptoms was associated with lower burden. These findings suggest that the transition from a previously healthy state to functional impairment may be associated with greater initial strain, while prolonged exposure to caregiving demands may coincide with reduced burden. Although the present study did not directly assess preparedness, coping strategies, perceived suddenness of onset, or expectation mismatch, the observed pattern is consistent with the possibility that adaptation over time moderates caregiver experience. Veterinary research on chronic disease management has similarly reported heightened owner distress when disease onset is unexpected or when caregiving responsibilities change abruptly, followed by improved coping as routines become established (28, 40, 44). Comparable patterns are well documented in human caregiving literature, where caregiver burden follows heterogeneous trajectories shaped by preparedness, symptom stability, and available support. Some caregivers experience stable or declining burden as they adapt to long-term care demands, while others experience persistently high or increasing burden when care demands intensify or support is limited (58–64). The present findings are consistent with the interpretation that caregiver burden in owners of dogs with chronic paresis or paralysis may reflect similar adaptive processes observed across veterinary and human caregiving contexts.

In the present study, employment in veterinary medicine was associated with significantly lower caregiver burden. Although preparedness, caregiving competence, and health literacy were not directly assessed, prior professional familiarity with clinical routines and disease management may contribute to greater confidence and perceived role clarity when providing care to a chronically impaired animal. Veterinary research on canine epilepsy has highlighted the substantial commitment required from owners managing this chronic neurological disorder and emphasizes the importance of expectation-setting and owner education in long-term care (20). Evidence from human caregiving research further demonstrates that higher levels of preparedness, caregiving competence, and education are associated with lower caregiver burden and depressive symptoms (65–69). In caregivers of patients with epilepsy, preparedness has been shown to be inversely related to burden, with higher educational level predicting lower strain (67). Similarly, preparedness has been identified as a protective factor against burden and depression in caregivers of individuals with physical disabilities, independent of caregiving duration (65). The present findings are consistent with the interpretation that prior professional exposure to medical environments may function as a buffering factor in the veterinary context. Familiarity with treatment processes, symptom management, and realistic expectations regarding disease progression may reduce uncertainty and perceived role strain. However, as constructs such as preparedness and caregiving competence were not measured in this study, this interpretation remains speculative and warrants direct examination in future research.

The present findings highlight caregiver burden as a relevant and potentially modifiable factor in the management of chronic neurological disease in dogs. Veterinary teams are well positioned to identify owners at risk of increased burden and to provide anticipatory guidance regarding long-term care demands. Recent veterinary literature emphasizes that support should extend beyond technical instruction and include acknowledgement of caregiver strain, empathic communication, and collaborative treatment planning to ensure that care routines are feasible within the owner's daily life (50). Simplifying treatment regimens where medically appropriate, proactively addressing practical challenges (e.g., mobility assistance, hygiene management, and environmental adaptations), and guiding structured problem-solving may strengthen caregiver confidence and reduce perceived burden. In addition, clear communication about therapeutic goals and expected outcomes can prevent frustration associated with perceived treatment inefficacy, which has been linked to higher burden (50). Encouraging social support and, when necessary, referral to allied mental health professionals may further mitigate strain. Addressing caregiver burden in a structured and collaborative manner may therefore improve owner wellbeing and support sustained, high-quality care for dogs with chronic paresis or paralysis.

Several limitations should be considered when interpreting these findings. First, the cross-sectional design does not permit conclusions regarding causal relationships between caregiver burden and psychosocial outcomes. Although strong associations were observed, the directionality of these relationships cannot be determined. Longitudinal research is needed to clarify trajectories of burden and potential adaptive processes over time.

Second, all variables were assessed using self-report instruments. While validated measures were applied, subjective reporting may be influenced by recall bias or shared method variance. However, caregiver burden is inherently a perceived construct, and self-report assessment is consistent with established human and veterinary caregiving research. In addition, owner-reported physical health complaints were assessed dichotomously (“yes/no”) without specification of symptom type or severity, limiting more detailed interpretation of the association between physical strain and caregiver burden. Furthermore, behavioral problems in the dogs were not assessed, and cannot be excluded in the control group, where they may have contributed to caregiver burden and potentially led to an underestimation of group differences.

Recruitment via online platforms may have introduced selection bias, and the predominance of female respondents may limit generalizability. Furthermore, the distribution of dog sizes differed between groups, with a higher proportion of large dogs in the healthy control group. Although dog size was not significantly associated with caregiver burden within the paresis/paralysis group, differences in body weight may nevertheless influence physical caregiving demands, particularly in conditions requiring lifting or mobility assistance. Future studies with more balanced group characteristics or adjusted analyses may help to further clarify the role of dog size in caregiving strain.

Finally, potentially relevant contextual factors such as financial strain, caregiving preparedness, and availability of external support were not assessed. Financial strain has been identified as an important contributor to psychological distress and caregiver burden (70). Psychosocial outcomes were only measured at a single time point, precluding evaluation of intra-individual change. Despite these limitations, the findings indicate that caregiver burden represents a meaningful component of chronic neurological disease management in dogs and warrants further investigation.

## 5 Conclusion

Owners of dogs with chronic paresis or paralysis experience significantly higher caregiver burden compared with owners of healthy dogs. In the present study, caregiver burden was associated with perceived practical impact on daily life, including negative effects on household cleanliness, physical strain, and lower owner-rated canine quality of life, whereas clinical severity parameters were not significantly related to burden. In addition, timing of symptom onset and duration of disease were associated with differences in burden levels.

These findings highlight caregiver burden as a relevant factor in the management of chronic neurological impairment in dogs. Addressing practical caregiving challenges and supporting owners in adapting to long-term care demands may help promote owner wellbeing and contribute to sustained, high-quality care for affected dogs.

## Data Availability

The datasets generated and analyzed during the current study are available from the corresponding author on reasonable request. Requests to access the datasets should be directed to Susanne Lauer, S.lauer@lmu.de.
