# Supplementary material for: Caregiver burden and psychosocial outcomes in owners of dogs with chronic paresis and paralysis compared with owners of healthy dogs
Source: Front Vet Sci. 2026 Jun 8;13:1837756. doi: 10.3389/fvets.2026.1837756 (PMC13283878; doi:10.3389/fvets.2026.1837756)
Supplement: Supplementary file 1 [file Data_Sheet_1.pdf]

MUSTER

Kleintierklinik der LMU München

Contact person: Janine Pryjmak

Caregiver Burden in Owners of Dogs with Chronic

Bitte so markieren: ☐ ☒ ☐ ☐ ☐ Bitte verwenden Sie einen Kugelschreiber oder nicht zu starken Filzstift. Dieser Fragebogen wird maschinell erfasst.

Korrektur: ☐ ■ ☐ ☒ ☐ Bitte beachten Sie im Interesse einer optimalen Datenerfassung die links gegebenen Hinweise beim Ausfüllen.

Privacy Policy

Dear Participant,
Thank you for your interest in the research project *"Caregiver Burden in Owners of Dogs with Chronic Paresis or Paralysis"*
The following information is intended to:
- inform you about the research project and the processing of personal data,
- explain your rights, and
- request your consent to participate.

Please take your time to read the information carefully. If you have any questions or require clarification, you are welcome to contact the primary investigator listed in the study information sheet. If you decide to participate in this research project, please confirm your consent at the end of this document.
Thank you for your support and trust in our work.

Kind regards,
Janine Pryjmak

1. General Information
This study aims to evaluate caregiver burden in owners of dogs with chronic paresis or paralysis. The findings are intended to contribute to improved counseling strategies for veterinarians, with the long-term goal of reducing caregiver burden in owners of paretic/paralyzed dogs. The Small Animal Clinic of the Ludwig-Maximilians-University Munich is involved in this research project. Data are collected using a questionnaire consisting of several sections, including a general information section and five validated questionnaires. Your task is to answer all questions honestly and completely so that your responses can be included in the study.

2. Details on Data Processing in the Research Project
This questionnaire is divided into eight sections. The majority consists of validated questionnaires, which are described below. The first, general section includes questions regarding your age, gender, occupation, the number of people in your household, and how many individuals are involved in caring for the pet. In addition, information regarding your cohabitation with the dog is collected. The Zarit Burden Interview, which constitutes the second part of the questionnaire, assesses caregiver burden. This instrument has been adapted for use in veterinary medicine and consists of 18 items, which are rated subjectively on a 5-point Likert scale ranging from "Never" to "Nearly always." The Perceived Stress Scale is a widely used measure of perceived stress. This 10-item scale assesses the degree of current stress and the extent to which life is experienced as unpredictable or overwhelming, using a 5-point scale ranging from "Never" to "Very often." The presence of depressive symptoms is assessed using the Center for Epidemiologic Studies Depression Scale. This 20-item instrument evaluates various symptoms and manifestations of depression, which are rated on a 4-point scale ranging from "Rarely or never" to "Most or all of the time." Anxiety symptoms are measured using the Generalized Anxiety Disorder 7-Item Scale, which comprises seven items rated on a 4-point scale ranging from "Not at all" to "Nearly every day." Quality of life is assessed using the Quality of Life Enjoyment and Satisfaction Questionnaire, a 16-item scale that measures the degree of enjoyment and satisfaction across various domains of daily life, including mood, health, work, and interpersonal relationships. Your data are processed in accordance with the General Data Protection Regulation (GDPR), the Bavarian Data Protection Act (BayDSG), and other applicable data protection regulations. Data processing is based on your consent pursuant to Article 6(1)(a) GDPR. For scientific analysis, all data are anonymized. The scientific evaluation of the data you provide is conducted at the Ludwig-Maximilians-Universität München. The duration of data storage is determined in accordance with statutory requirements. The data (including personal data) are stored locally on servers of the Ludwig-Maximilians-Universität München and are deleted as soon as and to the extent that they are no longer required for the stated processing purposes and are not subject to further statutory retention obligations. For scientific reasons, anonymized data (without personal identifiers) may be stored for up to ten years after publication of the study. The data are not shared with third parties. Your personal data are not used for automated decision-making, including profiling, in accordance with Article 22(1) and (4) GDPR.

3. Legal Basis for Data Processing
The legal basis for data processing is your consent (Article 6(1)(a) GDPR). Participation in this research project is voluntary. Providing your personal data is neither legally nor contractually required, nor is it necessary for the conclusion of a contract. Declining participation or withdrawing your consent will not result in any disadvantages. Withdrawal of consent applies only to future data processing; the lawfulness of data processing carried out on the basis of your consent prior to withdrawal remains unaffected. Please note that even in the event of withdrawal of consent, further data processing may remain permissible on the basis of another legal provision (cf. Article 17(1)(b) and Article 17(3)(d) GDPR), for example where the time-limited retention of raw data is necessary to ensure the verifiability of published research findings and compliance with principles of good scientific practice.

MUSTER

Privacy Policy [Fortsetzung]

4. Responsible Party and Contact Details
The research project is conducted by Janine Pryjmak, doctoral candidate at the Small Animal Clinic of the Ludwig-Maximilians-Universität München, under the supervision of Prof. Dr. med. vet. Susanne Lauer. If you have any questions regarding the project or data processing, if you wish to withdraw your consent, or if you would like to exercise any other rights under the General Data Protection Regulation (GDPR), please contact the following primary contact person in the first instance:
- Janine Pryjmak, Kleintierklinik der Ludwig-Maximilians-Universität München, Veterinärstr. 13, D-80539 München, E-Mail: janine.pryjmak@campus.lmu.de

The data controller within the meaning of Article 4(7) GDPR is:
- Janine Maria Pryjmak, Bauernfeindstraße 6, D-80939 München, E-Mail: janine.pryjmak@campus.lmu.de

5. Your Rights Under the General Data Protection Regulation (GDPR)
Under the General Data Protection Regulation (GDPR, Regulation (EU) 2016/679), you are entitled to the following rights: You have the right to request information as to whether and, if applicable, which personal data concerning you are being processed and to receive further information related to such processing (Article 15 GDPR). Please note that this right of access may be restricted or excluded in certain cases (see, in particular, Article 10 of the Bavarian Data Protection Act, BayDSG). If inaccurate personal data are being processed, you have the right to request rectification (Article 16 GDPR). Where the statutory requirements are met, you may request the erasure of your personal data or the restriction of their processing (Articles 17 and 18 GDPR). However, the right to erasure pursuant to Article 17(1) and (2) GDPR does not apply, among other cases, if the processing of personal data is necessary for the performance of a task carried out in the public interest or in the exercise of official authority (Article 17(3)(b) GDPR) or if statutory retention obligations apply. You have the right to receive the data you have provided in a structured, commonly used, and machine-readable format, or to have those data transmitted to a recipient designated by you, where the processing is based on consent or a contract and is carried out by automated means (Article 20 GDPR). You also have the right to withdraw your consent at any time with effect for the future (Article 7(3) GDPR). Withdrawal of consent does not affect the lawfulness of processing carried out on the basis of consent prior to withdrawal. Please note that even in the event of withdrawal of consent, further data processing may remain permissible on the basis of another legal provision (see Article 17(1)(b) and Article 17(3)(d) GDPR). In addition, you have the right to lodge a complaint with a supervisory authority within the meaning of Article 51 GDPR regarding the processing of your personal data. The competent supervisory authority for Ludwig-Maximilians-Universität München is the Bavarian State Commissioner for Data Protection, reachable at Postfach 22 12 19, 80502 Munich, Germany, or via https://www.datenschutz-bayern.de/service/complaint.html. Prior to submitting a complaint, the responsible organizational unit (Section 4) should be contacted in any case, as this ensures the fastest possible handling of your request. If you wish to exercise any of the above rights or have any questions, you may contact the department conducting the research project at any time using the contact details provided. We will then promptly review whether the legal requirements are met and take the necessary measures. Further restrictions, modifications, or exclusions of the above rights may arise from the GDPR or national legal provisions, for example where the exercise of these rights is likely to render the achievement of the research objectives impossible or seriously impair them and where such restriction is necessary for the fulfillment of the research purposes (see Article 25(4) BayDSG).

Declaration of consent

- [ ] I have received and read the information sheet dated January 18, 2024, on the Caregiver Burden research project and consent to participate in this research project and to the associated processing of my personal data. I am aware that I can refuse to participate and that non-participation in the research project will not result in any disadvantages. I have been expressly informed of the possibility of revoking my consent.

Inclusion Criteria

MUSTER

Inclusion Criteria [Fortsetzung]

To ensure the most unbiased possible study results, please confirm all of the following statements.
The response option regarding your mental health status is required because, without this information, the assumptions underlying the validated questionnaires would not be met, which could lead to biased survey results.

- ☐ The dog has been living in my care for at least six months.
- ☐ My dog does not exhibit one or more of the following symptoms and does not suffer from any of the following conditions: neurological symptoms, weakness, chronic or recurrent diarrhea, frequent or chronic vomiting, respiratory disease, or endocrine disorders.
- ☐ My dog does not receive any long-term medication (dietary supplements do not count as long-term medication).
- ☐ I am not currently receiving, and have not previously received, treatment for a mental health condition.

General Questions

Which of the following age categories do you belong to?
☐ 18 years or younger ☐ 19-29 years ☐ 30-39 years
☐ 40-49 years ☐ 50-59 years ☐ 60 years or older

Which gender do you identify with?
☐ Male ☐ Female ☐ Diverse
☐ Prefer not to say

What is your current marital status?
☐ Married ☐ Widowed ☐ Divorced
☐ Separated ☐ Single

What is your occupation?

How many people currently live in your household?
☐ One ☐ Two ☐ Three
☐ Four ☐ Five and more

How many people take care of the dog?
☐ One ☐ Two ☐ Three
☐ Four and more

How many animals are part of your household?
☐ One ☐ Two ☐ Three
☐ Four ☐ Five and more

What breed is your dog?

What gender is your dog?
☐ Female ☐ Female spayed ☐ Male
☐ Male neutered

How old is your four-legged friend?

How much does your dog weigh approximately?

How long have you owned your dog?

MUSTER

General Questions [Fortsetzung]

What kind of sporting activities do you enjoy with your dog?

- ☐ Daily walks
- ☐ Jogging
- ☐ Hiking
- ☐ Agility training
- ☐ Mantrailing
- ☐ Other sports

How would you rate your dog's quality of life? Very good ☐ ☐ ☐ ☐ ☐ Very poorly

Zarit Burden Interview

Question			
Do you feel that because of the time you spend with your pet that you don't have enough time for yourself?	☐ Never	☐ Rarely	☐ Sometimes
	☐ Quiet frequently	☐ Nearly always	
Do you feel stressed between caring for your pet and trying to meet other responsibilities for your family or work?	☐ Never	☐ Rarely	☐ Sometimes
	☐ Quite frequently	☐ Nearly always	
Do you feel angry when you are around your pet?	☐ Never	☐ Rarely	☐ Sometimes
	☐ Quite frequently	☐ Nearly always	
Do you feel that your pet currently affects our relationships with other family members or friends in a negative way?	☐ Never	☐ Rarely	☐ Sometimes
	☐ Quiet frequently	☐ Nearly always	
Are you afraid what the future holds for your pet?	☐ Never	☐ Rarely	☐ Sometimes
	☐ Quite frequently	☐ Nearly always	
Do you feel your pet is dependent on you?	☐ Never	☐ Rarely	☐ Sometimes
	☐ Quiet frequently	☐ Nearly always	
Do you feel strained when you are around your pet?	☐ Never	☐ Rarely	☐ Sometimes
	☐ Quiet frequently	☐ Nearly always	
Do you feel your health has suffered because of your involvement with your pet?	☐ Never	☐ Rarely	☐ Sometimes
	☐ Quiet frequently	☐ Nearly always	
Do you feel that you don't have as much privacy as you would like because of your pet?	☐ Never	☐ Rarely	☐ Sometimes
	☐ Quiet frequently	☐ Nearly always	
Do you feel that your social life has suffered because you are caring for your pet?	☐ Never	☐ Rarely	☐ Sometimes
	☐ Quiet frequently	☐ Nearly always	
Do you feel uncomfortable about having friends over because of your pet?	☐ Never	☐ Rarely	☐ Sometimes
	☐ Quiet frequently	☐ Nearly always	
Do you feel that you don't have enough money to take care of your pet in addition to the rest of your expenses?	☐ Never	☐ Rarely	☐ Sometimes
	☐ Quiet frequently	☐ Nearly always	
Do you feel that you will be unable to take care of your pet much longer?	☐ Never	☐ Rarely	☐ Sometimes
	☐ Quiet frequently	☐ Nearly always	
Do you feel you have lost control of your life since your pet's adoption?	☐ Never	☐ Rarely	☐ Sometimes
	☐ Quiet frequently	☐ Nearly always	
Do you wish you could leave the care of your pet to someone else?	☐ Never	☐ Rarely	☐ Sometimes
	☐ Quiet frequently	☐ Nearly always	
Do you feel you should be doing more for your pet?	☐ Never	☐ Rarely	☐ Sometimes
	☐ Quiet frequently	☐ Nearly always	
Do you feel you could do a better job in caring for your pet?	☐ Never	☐ Rarely	☐ Sometimes
	☐ Quiet frequently	☐ Nearly always	
Overall, how burdened do you feel in caring for your pet?	☐ Never	☐ Rarely	☐ Sometimes
	☐ Quiet frequently	☐ Nearly always	

Perceived Stress Scale (PSS)

Question			
In the last month, how often have you been upset because of something that happened unexpectedly?	☐ Never	☐ Almost never	☐ Sometimes
	☐ Fairly often	☐ Very often	
In the last month, how often have you felt that you were unable to control the important things in your life?	☐ Never	☐ Almost never	☐ Sometimes
	☐ Fairly often	☐ Very often	
In the last month, how often have you felt nervous and stressed?	☐ Never	☐ Almost never	☐ Sometimes
	☐ Fairly often	☐ Very often	
In the last month, how often have you felt confident about your ability to handle your personal problems?	☐ Never	☐ Almost never	☐ Sometimes
	☐ Fairly often	☐ Very often	
In the last month, how often have you felt that things were going your way?	☐ Never	☐ Almost never	☐ Sometimes
	☐ Fairly often	☐ Very often	

Perceived Stress Scale (PSS) [Fortsetzung]

In the last month, how often have you found that you could not cope with all the things that you had to do?	☐ Never<br>☐ Fairly often	☐ Almost never<br>☐ Very often	☐ Sometimes
In the last month, how often have you been able to control irritations in your life?	☐ Never<br>☐ Fairly often	☐ Almost never<br>☐ Very often	☐ Sometimes
In the last month, how often have you felt that you were on top of things?	☐ Never<br>☐ Fairly often	☐ Almost never<br>☐ Very often	☐ Sometimes
In the last month, how often have you been angered because of things that happened that were outside of your control?	☐ Never<br>☐ Fairly often	☐ Almost never<br>☐ Very often	☐ Sometimes
In the last month, how often have you felt difficulties were piling up so high that you could not overcome them?	☐ Never<br>☐ Fairly often	☐ Almost never<br>☐ Very often	☐ Sometimes

Center of Epidemiologic Studies Depression scale

My appetite was poor.	☐ Not at all / Less than 1 day<br>☐ 5-7 days in the past week	☐ 1-2 days in the past week<br>☐ Nearly every day for 2 weeks	☐ 3-4 days in the past week
I could not shake off the blues.	☐ Not at all / Less than 1 day<br>☐ 5-7 days in the past week	☐ 1-2 days in the past week<br>☐ Nearly every day for 2 weeks	☐ 3-4 days in the past week
I had trouble keeping my mind on what I was doing.	☐ Not at all / Less than 1 day<br>☐ 5-7 days in the past week	☐ 1-2 days in the past week<br>☐ Nearly every day for 2 weeks.	☐ 3-4 days in the past week
I felt depressed.	☐ Not at all / Less than 1 day<br>☐ 5-7 days in the past week	☐ 1-2 days in the past week<br>☐ Nearly every day for 2 weeks	☐ 3-4 days in the past week
My sleep was restless.	☐ Not at all / Less than 1 day<br>☐ 5-7 days in the past week	☐ 1-2 days in the past week<br>☐ Nearly every day for 2 weeks	☐ 3-4 days in the past week
I felt sad.	☐ Not at all / Less than 1 day<br>☐ 5-7 days in the past week	☐ 1-2 days in the past week<br>☐ Nearly every day for 2 weeks	☐ 3-4 days in the past week
I could not get going.	☐ Not at all / Less than 1 day<br>☐ 5-7 days in the past week	☐ 1-2 days in the past week<br>☐ Nearly every day for 2 weeks	☐ 3-4 days in the past week
Nothing made me happy.	☐ Not at all / Less than 1 day<br>☐ 5-7 days in the past week	☐ 1-2 days in the past week<br>☐ Nearly every day for 2 weeks	☐ 3-4 days in the past week
I felt like a bad person.	☐ Not at all / Less than 1 day<br>☐ 5-7 days in the past week	☐ 1-2 days in the past week<br>☐ Nearly every day for 2 weeks	☐ 3-4 days in the past week
I lost interest in my usual activities.	☐ Not at all / Less than 1 day<br>☐ 5-7 days in the past week	☐ 1-2 days in the past week<br>☐ Nearly every day for 2 weeks	☐ 3-4 days in the past week
I slept much more than usual.	☐ Not at all / Less than 1 day<br>☐ 5-7 days in the past week	☐ 1-2 days in the past week<br>☐ Nearly every day for 2 weeks	☐ 3-4 days in the past week

MUSTER

Center of Epidemiologic Studies Depression scale [Fortsetzung]

I felt like I was moving too slowly.	☐ Not at all / Less than 1 day<br>☐ 5-7 days in the past week	☐ 1-2 days in the past week<br>☐ Nearly every day for 2 weeks	☐ 3-4 days in the past week
I felt fidgety.	☐ Not at all / Less than 1 day<br>☐ 5-7 days in the past week	☐ 1-2 days in the past week<br>☐ Nearly every day for 2 weeks	☐ 3-4 days in the past week
I was tired all the time.	☐ Not at all / Less than 1 day<br>☐ 5-7 days in the past week	☐ 1-2 days in the past week<br>☐ Nearly every day for 2 weeks	☐ 3-4 days in the past week
I did not like myself.	☐ Not at all / Less than 1 day<br>☐ 5-7 days in the past week	☐ 1-2 days in the past week<br>☐ Nearly every day for 2 weeks	☐ 3-4 days in the past week
I lost a lot of weight without trying to.	☐ Not at all / Less than 1 day<br>☐ 5-7 days in the past week	☐ 1-2 days in the past week<br>☐ Nearly every day for 2 weeks	☐ 3-4 days in the past week
I had a lot of trouble getting to sleep.	☐ Not at all / Less than 1 day<br>☐ 5-7 days in the past week	☐ 1-2 days in the past week<br>☐ Nearly every day for 2 weeks	☐ 3-4 days in the past week
I could not focus on the important things.	☐ Not at all / Less than 1 day<br>☐ 5-7 days in the past week	☐ 1-2 days in the past week<br>☐ Nearly every day for 2 weeks	☐ 3-4 days in the past week
I wanted to hurt myself.	☐ Not at all / Less than 1 day<br>☐ 5-7 days in the past week	☐ 1-2 days in the past week<br>☐ Nearly every day for 2 weeks	☐ 3-4 days in the past week
I wished I were dead.	☐ Not at all / Less than 1 day<br>☐ 5-7 days in the past week	☐ 1-2 days in the past week<br>☐ Nearly every day for 2 weeks	☐ 3-4 days in the past week

Generalized Anxiety Disorder 7-Item Scale (GAD-7)

Over the last two weeks, how often have you been bothered by the following problems?

Feeling nervous, anxious, or on edge	☐ Not at all<br>☐ Nearly every day	☐ Several days	☐ More than half the days
Not being able to stop or control worrying	☐ Not at all<br>☐ Nearly every day	☐ Several days	☐ More than half the days
Worrying too much about different thrings	☐ Not at all<br>☐ Nearly every day	☐ Several days	☐ More than half the days
Trouble relaxing	☐ Not at all<br>☐ Nearly every day	☐ Several days	☐ More than half the days
Being so restless that it is hard to sit still	☐ Not at all<br>☐ Nearly every day	☐ Several days	☐ More than half the days
Becoming easily annoyed or irritable	☐ Not at all<br>☐ Nearly every day	☐ Several days	☐ More than half the days

MUSTER

MUSTER

Generalized Anxiety Disorder 7-Item Scale (GAD-7) [Fortsetzung]

Feeling afraid, as if something awful might happen	☐ Not at all	☐ Several days	☐ More than half the days
	☐ Nearly every day		

Quality of Life Enjoyment and Satisfaction Questionnaire (QLES-QSF) - Short

Taking everything into consideration, during the past week how satisfied have you been with your...

... physical health?	☐ Very poor	☐ Poor	☐ Fair
	☐ Good	☐ Very good	
... mood?	☐ Very poor	☐ Poor	☐ Fair
	☐ Good	☐ Very good	
... work?	☐ Very poor	☐ Poor	☐ Fair
	☐ Good	☐ Very good	
... household activities?	☐ Very poor	☐ Poor	☐ Fair
	☐ Good	☐ Very good	
... social relationships?	☐ Very poor	☐ Poor	☐ Fair
	☐ Good	☐ Very good	
... family relationships?	☐ Very poor	☐ Poor	☐ Fair
	☐ Good	☐ Very good	
... leisure time activities?	☐ Very poor	☐ Poor	☐ Fair
	☐ Good	☐ Very good	
... ability to function in daily life?	☐ Very poor	☐ Poor	☐ Fair
	☐ Good	☐ Very good	
... sexual drive, interest and/or performance?	☐ Very poor	☐ Poor	☐ Fair
	☐ Good	☐ Very good	
... economic status?	☐ Very poor	☐ Poor	☐ Fair
	☐ Good	☐ Very good	
... living/housing situation?	☐ Very poor	☐ Poor	☐ Fair
	☐ Good	☐ Very good	
... ability to get around physically without feeling dizzy or unsteady or falling?	☐ Very poor	☐ Poor	☐ Fair
	☐ Good	☐ Very good	
... your vision in terms of ability to do work or hobbies?	☐ Very poor	☐ Poor	☐ Fair
	☐ Good	☐ Very good	
... overall sense of well being?	☐ Very poor	☐ Poor	☐ Fair
	☐ Good	☐ Very good	
... medication? (If you don't take any, just skip the question)	☐ Very poor	☐ Poor	☐ Fair
	☐ Good	☐ Very good	
How would you rate your overall Life satisfaction and contentment during the past week?	☐ Very poor	☐ Poor	☐ Fair
	☐ Gut	☐ Very good	

Your personal experiences

I would be delighted if you would share your personal experiences or similar stories with me. You are welcome to do so using the field below, by email, or in person by telephone. If you decide to contact me by email or telephone, please note that you will no longer be anonymous.

MUSTER

MUSTER

Your personal experiences [Fortsetzung]

Thank you for participating in this survey!
Your responses have been saved.

MUSTER
