# Supplementary material for: Caregiver burden and psychosocial outcomes in owners of dogs with chronic paresis and paralysis compared with owners of healthy dogs
Source: Front Vet Sci. 2026 Jun 8;13:1837756. doi: 10.3389/fvets.2026.1837756 (PMC13283878; doi:10.3389/fvets.2026.1837756)
Supplement: Supplementary file 2 [file Data_Sheet_2.pdf]

evasys	Caregiver Burden	evasys

LMU Small Animal Clinic	Contact person: Janine Pryjmak<br>Caregiver Burden in Owners of Dogs with Chronic

Bitte so markieren: ☐ ☒ ☐ ☐ ☐ Bitte verwenden Sie einen Kugelschreiber oder nicht zu starken Filzstift. Dieser Fragebogen wird maschinell erfasst.
Korrektur: ☐ ■ ☐ ☒ ☐ Bitte beachten Sie im Interesse einer optimalen Datenerfassung die links gegebenen Hinweise beim Ausfüllen.

Privacy Policy

Dear Participant,

Thank you for your interest in the research project "*Caregiver Burden in Owners of Dogs with Chronic Paresis or Paralysis*"
The following information is intended to:
- inform you about the research project and the processing of personal data,
- explain your rights, and
- request your consent to participate.

Please take your time to read the information carefully. If you have any questions or require clarification, you are welcome to contact the primary investigator listed in the study information sheet. If you decide to participate in this research project, please confirm your consent at the end of this document. Thank you for your support and trust in our work.

Kind regards,
Janine Pryjmak

1. General Information
This study aims to evaluate caregiver burden in owners of dogs with chronic paresis or paralysis. The findings are intended to contribute to improved counseling strategies for veterinarians, with the long-term goal of reducing caregiver burden in owners of paretic/paralyzed dogs. The Small Animal Clinic of the Ludwig-Maximilians-University Munich is involved in this research project. Data are collected using a questionnaire consisting of several sections, including a general information section and five validated questionnaires. Your task is to answer all questions honestly and completely so that your responses can be included in the study.

2. Details on Data Processing in the Research Project
This questionnaire is divided into eight sections. The majority consists of validated questionnaires, which are described below. The first, general section includes questions regarding your age, gender, occupation, the number of people in your household, and how many individuals are involved in caring for the pet. In addition, information regarding your cohabitation with the dog is collected. The Zarit Burden Interview, which constitutes the second part of the questionnaire, assesses caregiver burden. This instrument has been adapted for use in veterinary medicine and consists of 18 items, which are rated subjectively on a 5- point Likert scale ranging from "Never" to "Nearly always." The Perceived Stress Scale is a widely used measure of perceived stress. This 10-item scale assesses the degree of current stress and the extent to which life is experienced as unpredictable or overwhelming, using a 5-point scale ranging from "Never" to "Very often." The presence of depressive symptoms is assessed using the Center for Epidemiologic Studies Depression Scale. This 20-item instrument evaluates various symptoms and manifestations of depression, which are rated on a 4-point scale ranging from "Rarely or never" to "Most or all of the time." Anxiety symptoms are measured using the Generalized Anxiety Disorder 7-Item Scale, which comprises seven items rated on a 4-point scale ranging from "Not at all" to "Nearly every day." Quality of life is assessed using the Quality of Life Enjoyment and Satisfaction Questionnaire, a 16-item scale that measures the degree of enjoyment and satisfaction across various domains of daily life, including mood, health, work, and interpersonal relationships. Your data are processed in accordance with the General Data Protection Regulation (GDPR), the Bavarian Data Protection Act (BayDSG), and other applicable data protection regulations. Data processing is based on your consent pursuant to Article 6(1)(a) GDPR. For scientific analysis, all data are anonymized. The scientific evaluation of the data you provide is conducted at the Ludwig-Maximilians-Universität München. The duration of data storage is determined in accordance with statutory requirements. The data (including personal data) are stored locally on servers of the Ludwig-Maximilians-Universität München and are deleted as soon as and to the extent that they are no longer required for the stated processing purposes and are not subject to further statutory retention obligations. For scientific reasons, anonymized data (without personal identifiers) may be stored for up to ten years after publication of the study. The data are not shared with third parties. Your personal data are not used for automated decision-making, including profiling, in accordance with Article 22(1) and (4) GDPR.

3. Legal Basis for Data Processing
The legal basis for data processing is your consent (Article 6(1)(a) GDPR). Participation in this research project is voluntary. Providing your personal data is neither legally nor contractually required, nor is it necessary for the conclusion of a contract. Declining participation or withdrawing your consent will not result in any disadvantages. Withdrawal of consent applies only to future data processing; the lawfulness of data processing carried out on the basis of your consent prior to withdrawal remains unaffected. Please note that even in the event of withdrawal of consent, further data processing may remain permissible on the basis of another legal provision (cf. Article 17(1)(b) and Article 17(3)(d) GDPR), for example where the time-limited retention of raw data is necessary to ensure the verifiability of published research findings and compliance with principles of good scientific practice.

Privacy Policy [Fortsetzung]

4. Responsible Party and Contact Details
The research project is conducted by Janine Pryjmak, doctoral candidate at the Small Animal Clinic of the Ludwig-Maximilians-Universität München, under the supervision of Prof. Dr. med. vet. Susanne Lauer. If you have any questions regarding the project or data processing, if you wish to withdraw your consent, or if you would like to exercise any other rights under the General Data Protection Regulation (GDPR), please contact the following primary contact person in the first instance: - Janine Pryjmak, Kleintierklinik der Ludwig-Maximilians-Universität München, Veterinärstr. 13, D-80539 München, E- Mail: janine.pryjmak@campus.lmu.de
The data controller within the meaning of Article 4(7) GDPR is:
- Janine Maria Pryjmak, Bauernfeindstraße 6, D-80939 München, E-Mail: janine.pryjmak@campus.lmu.de

5. Your Rights Under the General Data Protection Regulations (GDPR)
Under the General Data Protection Regulation (GDPR, Regulation (EU) 2016/679), you are entitled to the following rights: You have the right to request information as to whether and, if applicable, which personal data concerning you are being processed and to receive further information related to such processing (Article 15 GDPR). Please note that this right of access may be restricted or excluded in certain cases (see, in particular, Article 10 of the Bavarian Data Protection Act, BayDSG). If inaccurate personal data are being processed, you have the right to request rectification (Article 16 GDPR). Where the statutory requirements are met, you may request the erasure of your personal data or the restriction of their processing (Articles 17 and 18 GDPR). However, the right to erasure pursuant to Article 17(1) and (2) GDPR does not apply, among other cases, if the processing of personal data is necessary for the performance of a task carried out in the public interest or in the exercise of official authority (Article 17(3)(b) GDPR) or if statutory retention obligations apply. You have the right to receive the data you have provided in a structured, commonly used, and machine-readable format, or to have those data transmitted to a recipient designated by you, where the processing is based on consent or a contract and is carried out by automated means (Article 20 GDPR). You also have the right to withdraw your consent at any time with effect for the future (Article 7(3) GDPR). Withdrawal of consent does not affect the lawfulness of processing carried out on the basis of consent prior to withdrawal. Please note that even in the event of withdrawal of consent, further data processing may remain permissible on the basis of another legal provision (see Article 17(1)(b) and Article 17(3)(d) GDPR). In addition, you have the right to lodge a complaint with a supervisory authority within the meaning of Article 51 GDPR regarding the processing of your personal data. The competent supervisory authority for Ludwig-Maximilians-Universität München is the Bavarian State Commissioner for Data Protection, reachable at Postfach 22 12 19, 80502 Munich, Germany, or via https://www.datenschutz-bayern.de/service/complaint.html. Prior to submitting a complaint, the responsible organizational unit (Section 4) should be contacted in any case, as this ensures the fastest possible handling of your request. If you wish to exercise any of the above rights or have any questions, you may contact the department conducting the research project at any time using the contact details provided. We will then promptly review whether the legal requirements are met and take the necessary measures. Further restrictions, modifications, or exclusions of the above rights may arise from the GDPR or national legal provisions, for example where the exercise of these rights is likely to render the achievement of the research objectives impossible or seriously impair them and where such restriction is necessary for the fulfillment of the research purposes (see Article 25(4) BayDSG).

Declaration of consent

- [ ] I have received and read the information sheet dated August 13, 2024, regarding the Caregiver Burden research project, and I consent to participating in this research project and to the associated processing of my personal data. I am aware that I may decline to participate and that my decision not to participate in the research project will not result in any disadvantages. I have been expressly informed of my right to withdraw my consent.

Inclusion Criteria

To ensure the study results are as accurate as possible, please read all of the following eligibility criteria carefully and then confirm that they apply to you. I need your response regarding your mental health status because otherwise the validated questionnaires’ requirements will not be met, which could lead to distorted survey results.
- I have owned the dog for at least six months.
- The chronic paralysis has been present for at least six months.
- My dog does not exhibit any of the following symptoms or suffer from any of the following conditions: epilepsy, weakness, chronic or recurrent diarrhea, frequent or chronic vomiting, respiratory diseases, hormonal disorders.
- I am not currently undergoing, nor have I ever undergone, treatment for a mental health condition.

- [ ] I have carefully read and understood the eligibility requirements for participating in the study and hereby confirm my agreement to them.

General Questions

General Questions [Fortsetzung]

Which of the following age categories do you belong to?
☐ 18 years or younger ☐ 19-29 years ☐ 30-39 years
☐ 40-49 years ☐ 50-59 years ☐ 60 years or older

Which gender do you identify with?
☐ Male ☐ Female ☐ Diverse
☐ Prefer not to say

What is your current marital status?
☐ Married ☐ Widowed ☐ Divorced
☐ Separated ☐ Single

What is your occupation?

How many people currently live in your household?
☐ One ☐ Two ☐ Three
☐ Four ☐ Five and more

How many people take care of the dog?
☐ One ☐ Two ☐ Three
☐ Four and more

How is your chronically paretic/paralyzed dog cared for while you are away (e.g., on vacation)?

How many animals are part of your household?
☐ One ☐ Two ☐ Three
☐ Four ☐ Five and more

What breed is your dog?

What gender is your dog?
☐ Female ☐ Female spayed ☐ Male
☐ Male neutered

How old is your dog?

How much does your dog weigh approximately?

How long have you owned your dog?

How old was your dog when the chronic paresis/paralysis began?

General Questions [Fortsetzung]

Why did chronic paresis/paralysis develop?
- ☐ Tumor
- ☐ Previous trauma (e.g., spinal fracture or dislocation)
- ☐ Congenital condition
- ☐ Meningomyelitis
- ☐ Herniated disc
- ☐ Degenerative spinal cord disease
- ☐ Unknown reason

My dog can...
- ☐ only stand with support.
- ☐ nur mit Unterstützung laufen.
- ☐ walk on his own, but he wobbles while doing so.
- ☐ walk on his own, but he often falls over while doing so.
- ☐ not move his legs or walk or stand on his own.

Was the dog already part of your household before the chronic paresis/paralysis set in?
- ☐ Yes
- ☐ No

If the dog was already in your care before the onset of chronic paralysis, what activities did you engage in with it?
- ☐ Daily walks
- ☐ Jogging
- ☐ Hiking
- ☐ Agility
- ☐ Mantrailing
- ☐ Other Sports
- ☐ Working dog activities

Since the symptoms of paralysis appeared, which activities that you used to do with your dog are you no longer able to do?

What is your living situation like?
- ☐ House
- ☐ Ground-floor apartment
- ☐ Apartment with an elevator
- ☐ Apartment on the 1st floor without an elevator
- ☐ Apartment on the 2nd floor without an elevator
- ☐ Apartment on the 3rd floor or higher without an elevator

Is your dog's paresis/paralysis having a negative impact on the cleanliness of your apartment or house?
No, not at all ☐ ☐ ☐ ☐ ☐ Yes, very

Does your dog suffer from urinary and/or fecal incontinence?
- ☐ Urinary incontinence
- ☐ Fecal incontinence
- ☐ Urinary and fecal incontinence
- ☐ No

Do you help your dog empty its bladder if it has urinary incontinence? If so, how many times a day?

How do you manage your dog’s potential fecal incontinence?

How often do you have to bathe or clean your dog due to fecal or urinary incontinence?

MUSTER

General Questions [Fortsetzung]

How often do you have to clean your apartment or house because of your dog’s fecal or urinary incontinence?

Does your dog tend to develop pressure sores or wounds due to chronic paresis/paralysis? ☐ Yes ☐ No

Do you use dog shoes or something similar to protect your dog's paws?

Do you use a cart for support? ☐ Yes ☐ No

How much did the cart cost?

How well does your dog tolerate the cart? Very good ☐ ☐ ☐ ☐ ☐ Very poor

On average, how many days a week does your dog use the cart?

On the days when you use a cart, how long and how often does your dog use it each day?

For what activities does your dog use the cart?

Does your dog experience skin irritation or sores from using the cart? Never ☐ ☐ ☐ ☐ ☐ Always

In which areas do the wounds appear?

Do you use a sliding bag for support? ☐ Yes ☐ No

MUSTER

General Questions [Fortsetzung]

How much did the slide bag cost?

How well does your dog tolerate the slip bag? Very good ☐ ☐ ☐ ☐ ☐ Very poor

On average, how many days a week does your dog use the slide?

On the days you use a slide bag, how long and how often does your dog use it each day?

In what kinds of activities does your dog use the slide bag?

Apart from a cart or a sliding bag, do you use any other aids in your daily life?

Will the dog be transported using one or more of the aforementioned modes of transportation?
- ☐ Stroller
- ☐ Bicycle trailer
- ☐ Cargo bike with box
- ☐ Bicycle with dog basket
- ☐ Other form of transport

Do you think that a cart/sling bag or other assistive devices make your dog’s life easier?
- ☐ Yes
- ☐ No

How often do you have to visit the veterinarian because of the chronic paresis/paralysis (or related conditions)?

Do you regularly take your dog to physical therapy? If so, how often?

General Questions [Fortsetzung]

How much do you estimate the annual costs associated with your dog’s chronic paralysis to be? (e.g., veterinary bills, physical therapy, cart)

Do you suffer from back pain or similar issues due to caring for your paretic/paralyzed dog?

The people around me have generally reacted positively to the fact that I live with a chronically paralyzed dog.	Completely true	☐	☐	☐	☐	☐	Not true
Strangers generally react positively when they see me walking my paralyzed dog.	Completely true	☐	☐	☐	☐	☐	Not true
How would you rate your dog’s quality of life since the onset of paresis/paralysis?	Very good	☐	☐	☐	☐	☐	Very poor

Zarit Burden Interview

Do you feel that because of the time you spend with your pet that you don’t have enough time for yourself?	☐ Never<br>☐ Quiet frequently	☐ Rarely<br>☐ Nearly always	☐ Sometimes
Do you feel stressed between caring for your pet and trying to meet other responsibilities for your family or work?	☐ Never<br>☐ Quiet frequently	☐ Rarely<br>☐ Nearly always	☐ Sometimes
Do you feel angry when you are around your pet?	☐ Never<br>☐ Quiet frequently	☐ Rarely<br>☐ Nearly always	☐ Sometimes
Do you feel that your pet currently affects your relationships with other family members or friends in a negative way?	☐ Never<br>☐ Quiet frequently	☐ Rarely<br>☐ Nearly always	☐ Sometimes
Are you afraid what the future holds for your pet?	☐ Never<br>☐ Quiet frequently	☐ Rarely<br>☐ Nearly always	☐ Sometimes
Do you feel your pet is dependent on you?	☐ Never<br>☐ Quiet frequently	☐ Rarely<br>☐ Nearly always	☐ Sometimes
Do you feel strained when you are around your pet?	☐ Never<br>☐ Quiet frequently	☐ Rarely<br>☐ Nearly always	☐ Sometimes
Do you feel your health has suffered because of your involvement with your pet?	☐ Never<br>☐ Quiet frequently	☐ Rarely<br>☐ Nearly always	☐ Sometimes
Do you feel that you don’t have as much privacy as you would like because of your pet?	☐ Never<br>☐ Quiet frequently	☐ Rarely<br>☐ Nearly always	☐ Sometimes
Do you feel that your social life has suffered because you are caring for your pet?	☐ Never<br>☐ Quiet frequently	☐ Rarely<br>☐ Nearly always	☐ Sometimes
Do you feel uncomfortable about having friends over because of your pet?	☐ Never<br>☐ Quiet frequently	☐ Rarely<br>☐ Nearly always	☐ Sometimes
Do you feel that you don’t have enough money to take care of your pet in addition to the rest of your expenses?	☐ Never<br>☐ Quiet frequently	☐ Rarely<br>☐ Nearly always	☐ Sometimes
Do you feel that you will be unable to take care of your pet much longer?	☐ Never<br>☐ Quiet frequently	☐ Rarely<br>☐ Nearly always	☐ Sometimes
Do you feel you have lost control of your life since your pet's paretic/paralyzed?	☐ Never<br>☐ Quiet frequently	☐ Rarely<br>☐ Nearly always	☐ Sometimes
Do you wish you could leave the care of your pet to someone else?	☐ Never<br>☐ Quiet frequently	☐ Rarely<br>☐ Nearly always	☐ Sometimes

MUSTER

Zarit Burden Interview [Fortsetzung]

Do you feel you should be doing more for your pet?	☐ Never<br>☐ Quiet frequently	☐ Rarely<br>☐ Nearly always	☐ Sometimes
Do you feel you could do a better job in caring for your pet?	☐ Never<br>☐ Quiet frequently	☐ Rarely<br>☐ Nearly always	☐ Sometimes
Overall, how burdened do you feel in caring for your pet?	☐ Never<br>☐ Quiet frequently	☐ Rarely<br>☐ Nearly always	☐ Sometimes

Perceived Stress Scale (PSS)

In the last month, how often have you been upset because of something that happened unexpectedly?	☐ Never<br>☐ Fairly often	☐ Almost never<br>☐ Very often	☐ Sometimes
In the last month, how often have you felt that you were unable to control the important things in your life?	☐ Never<br>☐ Fairly often	☐ Almost never<br>☐ Very often	☐ Sometimes
In the last month, how often have you felt nervous and stressed?	☐ Never<br>☐ Fairly often	☐ Almost never<br>☐ Very often	☐ Sometimes
In the last month, how often have you felt confident about your ability to handle your personal problems?	☐ Never<br>☐ Fairly often	☐ Almost never<br>☐ Very often	☐ Sometimes
In the last month, how often have you felt that things were going your way?	☐ Never<br>☐ Fairly often	☐ Almost never<br>☐ Very often	☐ Sometimes
In the last month, how often have you found that you could not cope with all the things that you had to do?	☐ Never<br>☐ Fairly often	☐ Almost never<br>☐ Very often	☐ Sometimes
In the last month, how often have you been able to control irritations in your life?	☐ Never<br>☐ Fairly often	☐ Almost never<br>☐ Very often	☐ Sometimes
In the last month, how often have you felt that you were on top of things?	☐ Never<br>☐ Fairly often	☐ Almost never<br>☐ Very often	☐ Sometimes
In the last month, how often have you been angered because of things that happened that were outside of your control?	☐ Never<br>☐ Fairly often	☐ Almost never<br>☐ Very often	☐ Sometimes
In the last month, how often have you felt difficulties were piling up so high that you could not overcome them?	☐ Never<br>☐ Fairly often	☐ Almost never<br>☐ Very often	☐ Sometimes

Center of Epidemiologic Studies Depression scale

My appetite was poor.	☐ Not at all / Less than 1 day<br>☐ 5-7 days in the past week	☐ 1-2 days in the past week<br>☐ Nearly every day for 2 weeks	☐ 3-4 days in the past week
I could not shake off the blues.	☐ Not at all / Less than 1 day<br>☐ 5-7 days in the past week	☐ 1-2 days in the past week<br>☐ Nearly every day for 2 weeks	☐ 3-4 days in the past week
I had trouble keeping my mind on what I was doing.	☐ Not at all / Less than 1 day<br>☐ 5-7 days in the past week	☐ 1-2 days in the past week<br>☐ Nearly every day for 2 weeks	☐ 3-4 days in the past week
I felt depressed.	☐ Not at all / Less than 1 day<br>☐ 5-7 days in the past week	☐ 1-2 days in the past week<br>☐ Nearly every day for 2 weeks	☐ 3-4 days in the past week
My sleep was restless.	☐ Not at all / Less than 1 day<br>☐ 5-7 days in the past week	☐ 1-2 days in the past week<br>☐ Nearly every day for 2 weeks	☐ 3-4 days in the past week
I felt sad.	☐ Not at all / Less than 1 day<br>☐ 5-7 days in the past week	☐ 1-2 days in the past week<br>☐ Nearly every day for 2 weeks	☐ 3-4 days in the past week

MUSTER

Center of Epidemiologic Studies Depression scale [Fortsetzung]

I could not get going.	☐ Not at all / Less than 1 day<br>☐ 5-7 days in the past week	☐ 1-2 days in the past week<br>☐ Nearly every day for 2 weeks	☐ 3-4 days in the past week
Nothing made me happy.	☐ Not at all / Less than 1 day<br>☐ 5-7 days in the past week	☐ 1-2 days in the past week<br>☐ Nearly every day for 2 weeks	☐ 3-4 days in the past week
I felt like a bad person.	☐ Not at all / Less than 1 day<br>☐ 5-7 days in the past week	☐ 1-2 days in the past week<br>☐ Nearly every day for 2 weeks	☐ 3-4 days in the past week
I lost interest in my usual activities.	☐ Not at all / Less than 1 day<br>☐ 5-7 days in the past week	☐ 1-2 days in the past week<br>☐ Nearly every day for 2 weeks	☐ 3-4 days in the past week
I slept much more than usual.	☐ Not at all / Less than 1 day<br>☐ 5-7 days in the past week	☐ 1-2 days in the past week<br>☐ Nearly every day for 2 weeks	☐ 3-4 days in the past week
I felt like I was moving too slowly.	☐ Not at all / Less than 1 day<br>☐ 5-7 days in the past week	☐ 1-2 days in the past week<br>☐ Nearly every day for 2 weeks	☐ 3-4 days in the past week
I felt fidgety.	☐ Not at all / Less than 1 day<br>☐ 5-7 days in the past week	☐ 1-2 days in the past week<br>☐ Nearly every day for 2 weeks	☐ 3-4 days in the past week
I was tired all the time.	☐ Not at all / Less than 1 day<br>☐ 5-7 days in the past week	☐ 1-2 days in the past week<br>☐ Nearly every day for 2 weeks	☐ 3-4 days in the past week
I did not like myself.	☐ Not at all / Less than 1 day<br>☐ 5-7 days in the past week	☐ 1-2 days in the past week<br>☐ Nearly every day for 2 weeks	☐ 3-4 days in the past week
I lost a lot of weight without trying to.	☐ Not at all / Less than 1 day<br>☐ 5-7 days in the past week	☐ 1-2 days in the past week<br>☐ Nearly every day for 2 weeks	☐ 3-4 days in the past week
I had a lot of trouble getting to sleep.	☐ Not at all / Less than 1 day<br>☐ 5-7 days in the past week	☐ 1-2 days in the past week<br>☐ Nearly every day for 2 weeks	☐ 3-4 days in the past week
I could not focus on the important things.	☐ Not at all / Less than 1 day<br>☐ 5-7 days in the past week	☐ 1-2 days in the past week<br>☐ Nearly every day for 2 weeks	☐ 3-4 days in the past week
I wanted to hurt myself.	☐ Not at all / Less than 1 day<br>☐ 5-7 days in the past week	☐ 1-2 days in the past week<br>☐ Nearly every day for 2 weeks	☐ 3-4 days in the past week
I wished I were dead.	☐ Not at all / Less than 1 day<br>☐ 5-7 days in the past week	☐ 1-2 days in the past week<br>☐ Nearly every day for 2 weeks	☐ 3-4 days in the past week

MUSTER

Generalized Anxiety Disorder 7-Item Scale (GAD-7)

Over the last two weeks, how often have you been bothered by the following problems?

Feeling nervous, anxious, or on edge	☐ Not at all<br>☐ Nearly every day	☐ Several days	☐ More than half the days
Not being able to stop or control worrying	☐ Not at all<br>☐ Nearly every day	☐ Several days	☐ More than half the days
Worrying too much about different thrings	☐ Not at all<br>☐ Nearly every day	☐ Several days	☐ More than half the days
Trouble relaxing	☐ Not at all<br>☐ Nearly every day	☐ Several days	☐ More than half the days
Being so restless that it is hard to sit still	☐ Not at all<br>☐ Nearly every day	☐ Several days	☐ More than half the days
Becoming easily annoyed or irritable	☐ Not at all<br>☐ Nearly every day	☐ Several days	☐ More than half the days
Feeling afraid, as if something awful might happen	☐ Not at all<br>☐ Nearly every day	☐ Several days	☐ More than half the days

Quality of Life Enjoyment and Satisfaction Questionnaire (QLES-QSF) - Short

Taking everything into consideration, during the past week how satisfied have you been with your...

... physical health?	☐ Very poor<br>☐ Good	☐ Poor<br>☐ Very good	☐ Fair
... work?	☐ Very poor<br>☐ Good	☐ Poor<br>☐ Very good	☐ Fair
... household activities?	☐ Very poor<br>☐ Good	☐ Poor<br>☐ Very good	☐ Fair
... social relationships?	☐ Very poor<br>☐ Good	☐ Poor<br>☐ Very good	☐ Fair
... family relationships?	☐ Very poor<br>☐ Good	☐ Poor<br>☐ Very good	☐ Fair
... leisure time activities?	☐ Very poor<br>☐ Good	☐ Poor<br>☐ Very good	☐ Fair
... ability to function in daily life?	☐ Very poor<br>☐ Good	☐ Poor<br>☐ Very good	☐ Fair
... sexual drive, interest and/or performance?	☐ Very poor<br>☐ Good	☐ Poor<br>☐ Very good	☐ Fair
... economic status?	☐ Very poor<br>☐ Good	☐ Poor<br>☐ Very good	☐ Fair
... living/housing situation?	☐ Very poor<br>☐ Good	☐ Poor<br>☐ Very good	☐ Fair
... ability to get around physically without feeling dizzy or unsteady or falling?	☐ Very poor<br>☐ Good	☐ Poor<br>☐ Very good	☐ Fair
... your vision in terms of ability to do work or hobbies?	☐ Very poor<br>☐ Good	☐ Poor<br>☐ Very good	☐ Fair
... overall sense of well being?	☐ Very poor<br>☐ Good	☐ Poor<br>☐ Very good	☐ Fair
... medication? (If you don't take any, just skip the question)	☐ Very poor<br>☐ Good	☐ Poor<br>☐ Very good	☐ Fair
How would you rate your overall Life satisfaction and contentment during the past week?	☐ Very poor<br>☐ Good	☐ Poor<br>☐ Very good	☐ Fair

Your personal experiences

MUSTER

MUSTER

Your personal experiences [Fortsetzung]

I would be delighted if you would share your personal experiences or similar stories with me. You are welcome to do so using the field below, by email, or in person by telephone. If you decide to contact me by email or telephone, please note that you will no longer be anonymous.

Thank you for participating in this survey!
Your responses have been saved.

MUSTER
